# Characteristic Features of Wound Dressings Based on Butyric-Acetic Chitin Copolyesters—Results of Clinical Trials

**DOI:** 10.3390/ma12244170

**Published:** 2019-12-12

**Authors:** Ilona Latańska, Anna Kozera-Żywczyk, Elwira Beata Paluchowska, Witold Owczarek, Andrzej Kaszuba, Marcin Noweta, Józef Tazbir, Beata Kolesińska, Zbigniew Draczyński, Witold Sujka

**Affiliations:** 1 Tricomed SA, Świętojańska Street 5/9, 93-493 Lodz, Poland; ilona.latanska@tricomed.com; 2 Military Institute of Medicine, Ministry of National Defense Central Clinical Hospital, Dermatology Clinic, Szaserów Street 128, 04-141 Warsaw, Poland; annakozera@o2.pl (A.K.-Ż.); epaluchowska@betiva.com (E.B.P.); witold.owczarek@dermedicus.pl (W.O.); 3 Wł. Biegański Provincial Specialised Hospital, Dermatology, Paediatric Dermatology and Oncologic Dermatology Ward, Dermatology, UM Paediatric Dermatology and Oncologic Dermatology Clinic, Kniaziewicza Street 1/5, 91-347 Lodz, Poland; andrzej.kaszuba@icloud.com (A.K.); m.noweta@wp.pl (M.N.); 4 Citonet Lodz Limited Company, Wound Treatment Clinic., Świętojańska Street 5/9, 93-493 Lodz, Poland; jozef.tazbir@op.pl; 5 Organic Chemistry Unit, Lodz University of Technology, Żeromskiego Street 116, 90-924 Lodz, Poland; beata.kolesinska@p.lodz.pl; 6 Institute of Material Science of Textiles and Polymer Composites, Lodz University of Technology, Żeromskiego Street 116, 90-924 Lodz, Poland; zbigniew.draczynski@p.lodz.pl

**Keywords:** clinical trials, butyric-acetic chitin copolyester, resorptive dressings, Medisorb R, silver

## Abstract

The article presents the results of clinical trials of wound dressings whose main ingredient is butyric-acetic chitin copolyester (BAC 90:10). It is a chitin derivative soluble in typical organic solvents. During the trial, the dressings were used on wounds resulting from venous insufficiency or diabetes. The trial evaluated the safety of use and efficacy of three forms of the dressing including porous membrane (Medisorb R Membrane), porous membrane with silver (Medisorb R Ag), and powder (Medisorb R Powder). The clinical trial had a multi-centre character. Three medical units were engaged in the study. The trial included 36 patients (12 men and 24 women). The mean age of the participants was 65 years of age (age range: 26–96). The choice of dressings was made on the basis of preliminary evaluation of the wound, clinical signs of infection, or risk of infection. Medisorb R Membrane dressing was used in 23 patients, Medisorb R Ag dressing was used in 15 patients, and Medisorb R powder was used in two patients. During the course of the trial, there were 10 control visits planned. The obtained results prove the safety and efficacy of dressings in question. The efficacy of treatment was evaluated as good. In the majority of patients, the ulceration was decreased both on the surface and in depth. The success of the treatment relied not only on the applied dressing, but also the stage of the basic disease, the accompanying diseases, and the age of the patient.

## 1. Introduction

Currently, there are many wound dressings available on the market, which are very useful in dressing both small wounds and the ones that are hard to heal. However, among this group, there are very few dressings that are biodegradable. Biodegradability is a very important feature of a dressing for wounds with a compromised healing process. In such wounds, a change of the dressing physically disrupts the structure of tissues in the process of regeneration, which results in longer healing time and prolonged convalescence of the patient [1]. This problem may be eliminated by the use of dressing materials, which are biodegradable and resorptive due to the action of enzymes present in tissues. There are a few biodegradable dressings whose main function is haemostasis. An example of such dressing may be Sorbsan, which is a dressing made of alginate fibres. Another dressing of this kind is Traumastem, which is made of oxidized cellulose. One of the raw materials, which may be used for manufacturing biodegradable dressings, is also chitin. There are many chitin derivatives characterized by features useful from the point of view of dressing wounds [2,3,4,5]. Chitin is mainly obtained from the shells of marine crustaceans, such as lobsters and crabs, where the content of chitin is from 20% to 40% [6,7]. There are also alternative sources of chitin, such as fungi or insects [8,9]. Chitin and its derivatives, besides being used in dressing materials, may be used as haemostats, dietary supplements, drug carriers, and more [10]. Biodegradation of chitin takes place due to the presence of different enzymes. It is known that, in the presence of human enzymes, such as chitotriosidase, which belongs to the group of chitinases and chitin biodegrades [11,12]. Due to the fact that chitin is an integral part of fungi, bacteria, and other harmful microorganisms’ cell walls, it is believed that the mammalian immune system, when in contact with chitin, produces antibodies responsible for identifying the molecular structure of pathogens [13]. Due to this, pathogens’ cell walls are destroyed, which leads to their deactivation and eliminates the risk of infection. This is the reason why, when chitin or chitin derivatives dressing materials are used, the human organism reacts to them during an infection. The process consists of several stages, which include recognizing chitin and releasing chitotriosidase that decomposes glycoside bonds. For chitin, the final degradation product is N-acetyl-D-glucosamine. N-acetyl-D-glucosamine is further broken down into derivatives, which the body uses, among others, for the synthesis of glycosaminoglycans (GAG). This can be a source of building material for new cells [14]. N-acetyl-D-glucosamine is, at the same time, a key component of the extracellular matrix (ECM), which mediates the transmission of signals between cells, and also takes part in the processes of migration and the growth of cells. Apart from glycosaminoglycans, other components of ECM also influence the water absorption and creation of gel of a significant volume, which later translates into high resistance of ECM to compression. N-acetyl-D-glucosamine is a component of cartilage matrix and, thus, has key importance in particular connections [15]. On the other hand, the properties of N-acetylglucosamine and glucosamine are widely used to treat arthritis. In humans, glucosamine and N-acetyl-D-glucosamine are also a building material for disaccharides included in glycosaminoglycans such as hyaluronic acid, chondroitin sulphate and keratan sulphate, which are necessary for the reconstruction and maintenance of cartilage and joints in a healthy condition. In order to prevent diseases of the mentioned tissues, patients experiencing problems with joints and elderly people are recommended to take dietary supplements containing glucosamine and its derivatives [16]. 

Chitin is a biocompatible substance, which influences activation of macrophages and increases the intensity of granulation tissue and proliferation of small blood vessels in the wound, which accelerates the wound-healing process [17]. However, the biggest obstacle in wide use of chitin is its nearly absolute lack of solubility in water and other water solvents. This feature prompted intense research projects aimed at discovering ester or copolyester chitin derivatives, which would be characterized by better solubility when compared to natural chitin.

There are many examples of research for obtaining dressings based on chitin or its derivatives in which the following properties of dressings were confirmed: biocompatibility, bactericidal activity, pain relief, and induction of tissue healing, which accelerates the wound healing process [18,19,20]. It was demonstrated that chitin copolyesters, such as di-butyryl-chitin (DBC), are non-toxic for fibroblasts and, after contact with DBC, the cells exhibit the ability to create new colonies. It was also demonstrated that those derivatives do not invoke irritation on animal skin. The research [21] on polypropylene meshes coated in chitin and DBC and their influence on the healing process showed that full-depth wounds dressed with such dressings healed faster and were more damp and elastic when compared to wounds dressed only with gauze. Moreover, the microscopic evaluation concluded that, after 10 days, the wound was filled with granulation fully covered with epithelium. After 14 days, the majority of wounds were filled with connective tissue with blood vessels, collagen, and elastic fibres. On the contrary, the wounds dressed only with gauze were characterized by long-term exudation (above 10 days) and their healing process was finished after 21 days. It was demonstrated that both DBC and chitin do not invoke the increase in TNF-α activity, interferon, and nitrogen oxide levels whose presence indicates the occurrence of the inflammatory process and disturbs the healing process. The comparative tests [22] of materials made of di-butyryl-chitin, Chitosan, and chitin showed that DBC, when compared with other materials, induces the growth of granulation within the wound. This fact is connected with the increase of glycosaminoglycans (GAG) in the regenerating tissues and increased the amount of created cells after the application of DBC. Due to the great potential related to the possibility of processing and the formation of different kinds of products out of chitin copolyesters, there were also different tests carried out that aimed at the determination of the influence of the applied dressing on healing processes. There were other comparative studies [23] of DBC dressings’ influence on the development of human keratinocytes. One group of dressings was made of non-woven fabric manufactured with electrospinning technology and the other was manufactured using traditional methods. The tests showed that both dressings accelerated wound healing. However, electrospun non-woven fabric accelerated the process of cell remodelling in the wound and the formation of type I collagen and filaggrin.

The clinical trials on humans, which have been carried out so far [24,25], also prove that DBC dressings in the form of non-woven fabric demonstrate high efficiency in treating different kinds of wounds. The healing time was significantly shortened. In some cases, it was also possible to eliminate the necessity for skin grafts. The dressings were placed on the wound and not changed until the wound was totally healed. 

The manufacturing process of non-woven DBC dressings comprises many stages such as the formation of fibres and non-woven applications or the creation of different forms of dressings with the use of different techniques and specialized devices. In order to simplify the manufacturing process (economic aspect), a porous membrane form of the dressing was developed. In pursuance of better mechanical properties of the dressing, some butyric groups were substituted by acetic ones (molar ratio of butyric groups to acetic ones being 9:1).

As part of the research, it was determined that the efficacy and safety of use of dressings based on butyric-acetic chitin copolyester (BAC 90:10) should be tested in clinical conditions. The dressings were supposed to be used in the treatment of chronic, hard-to-heal skin loss resulting from venous insufficiency ulceration and wounds resulting from diabetes. It was planned that the dressing based on the butyric-acetic chitin copolyester will be applied in three forms: Medisorb R Membrane–porous membrane, Medisorb R Ag–porous membrane impregnated with micro silver, and Medisorb R Powder–powdered dressing. As a reference, dressing an absorbent product was used. The Medisorb P Plus was fixed by the use of a bandage or Codofix/Codofix Plus dressing net. The dressings subjected to tests can be used for dressing chronic, hard-to-heal wounds resulting from venous diseases, diabetes, pressure ulcers, and post-operative wounds.

Crus ulceration is a condition where patients suffer from chronic, painful, and hard-to-heal wounds. An ulceration is a wound penetrating through the epidermis and reaching dermal layers. Most commonly, crus ulceration is caused by venous diseases encompassing the veins and arteries (85%) [26]. Venous ulcerations are chronic wounds requiring intense treatment. The wounds themselves have a significant bearing on the quality of patients’ lives. Inadequate treatment may result in deterioration of the wound condition. The bigger the ulceration and the longer its presence, the more complicated the healing process becomes [27]. There are many theories that attempt to explain the mechanisms of crus ulcer formation. Within the lower extremities, valve insufficiency causes venous hypertension, which can lead to tissue damage and ulceration [28]. An increase in venous pressure causes the expansion and elongation of the skin’s capillaries, which disrupts normal microcirculation [29,30]. The level of leukocytes increases as hypertension leads to the capture and activation of leukocytes, which releases free radicals and other toxic substances that promote cell death and tissue damage [31]. Hypertension in capillaries causes the penetration of macromolecules into the dermis, which stops growth factors and cytokines necessary for tissue repair [31]. There are also disturbances in the work of the fibrinolytic system, in which fibrin is usually quickly removed. The resulting disorders in oxygen and nutrient diffusion lead to tissue hypoxia, cell death, and ulceration [32]. The correct diagnosis of venous ulceration is extremely important. Clinical characteristics of venous ulcers include leg swelling, pain, discharge, and lipodermatosclerosis [33,34]. Complications associated with venous ulcers include changes in the manner of walking, pain, infections, cellulitis, or dermatitis [35]. 

Crus ulcers are most often caused by venous insufficiency, but may also result from arterial pathology (most often with atherosclerotic aetiology) and diabetes. Treatment of crus ulcers is very difficult and long-lasting. Thus, the prevention of leg ulcers and conservative therapy are of great importance. 

The choice of treatment and type of dressing should depend on the aetiology of the wound, its location and size, and the stage of the healing process. Compression therapy is the gold standard in treating leg ulcers. It increases local hydrostatic pressure and reduces the pressure in superficial veins, which reduces the formation of exudate. Gradual pressure accelerates blood flow within deep veins. This therapy also causes local release of plasminogen, which improves the conditions associated with local fibrinolysis and dissolution of fibrous deposits located within the ulcer. The most important goal of compression therapy is to prevent crus ulcers. In the treatment phase of venous insufficiency, i.e., in the acute phase of crus ulceration, the use of bandages is recommended. The best results are achieved by gradual pressure, which means that the greatest pressure is applied within the ankle area, and it is getting smaller toward the top. Pressure applied on the crus area causes a decrease in the flow through the superficial venous vessels. This is an increase in the flow through the deep vessels, which improves venous return and microcirculation [36].

General pharmacological treatment is also used to treat crus ulcers. In the process of treating leg ulcers, it is important to clean the wound from necrotic tissues and maintain a clean, moist environment in the wound [37].

One of the most important elements in the treatment of chronic wounds, such as crus ulcers due to venous insufficiency, is the use of appropriate dressings. Various agents are used to treat such wounds, e.g. sterile saline, hydrogel, iodopovidone solutions, hypochlorite acid, or collagenase. A superoxidized solution that has antimicrobial properties is also effective. A variety of dressings are also used such as highly absorbent foam dressings that maintain a moist environment around the wound as well as alginate, hydrofibre dressings, hydrocolloids, oxidized regenerated cellulose, or micronized collagen. Many of these dressings are available in an alternative version with the addition of silver [38].

Surgical treatment is usually used when other treatments fail.

A systematic approach to both wound assessment and treatment most often lead to favourable results. A multidisciplinary approach to wound care proves to be very effective and widely recommended [38].

Due to the analysis of current methods of treatment for difficult-to-heal wounds under the influence of the environment, it can be stated that the support of Medisorb R/ Medisorb R Ag dressings may shorten the convalescence process in a measurable way. Plasma devices with the closed air circle with a hydrogen peroxide additive sufficiently decreases the bacterial flora in the wound and its surroundings. Dressing synergic activity and the use of plasma, which does not react with tissue directly, but deactivates microorganisms, provides effective antibacterial activity during treatment, which joins the two methods [39]. It is similar to nonthermal plasma activity in direct contact with infected wound. Nonetheless, it is challenging to establish its influence on Medisorb R dressing and its parameters change [40]. Lowering the pressure in the wound neighbourhood (VAC- vacuum assisted closure) during the use of dressings on the basis of chitin co-polyesters may be beneficial [41]. However, drawing a clear conclusion from the use of combination therapy may be possible after in vivo tests of the method and dressing itself.

## 2. Materials and Methods 

The clinical trial evaluated the efficacy and safety of using the following products.

**Figure 1:**
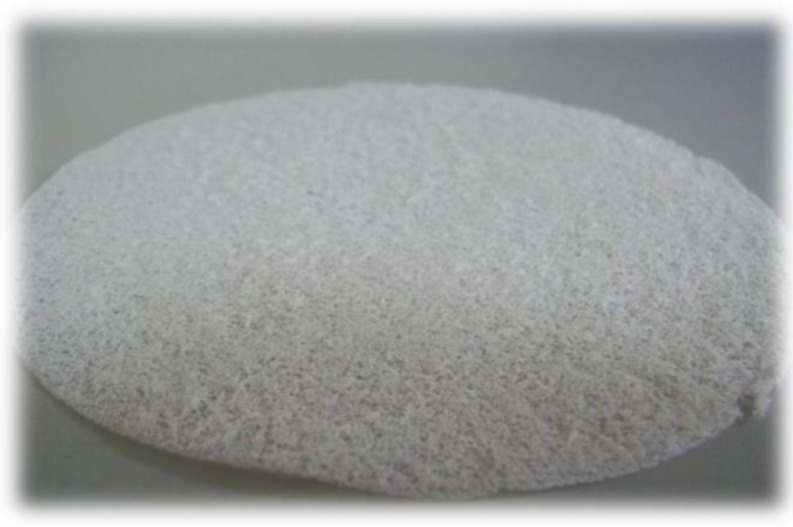
Medisorb R Membrane/Medisorb R Ag dressing.

**Figure 2:**
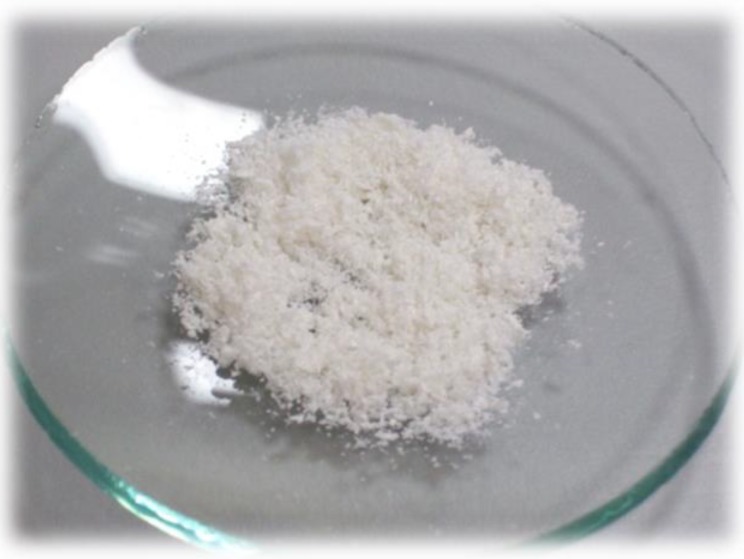
Medisorb R Powder dressing.

- Medisorb R Membrane—a dressing in the form of a porous membrane, which, in its structure, contains microcapillaries that evacuate the exudate from the wound (Figure 1).
- Medisorb R Ag—a dressing in the form of a porous membrane with micro silver which, similar to Medisorb R, contains in its structure microcapillaries. The dressing has bactericidal and bacteriostatic properties. It is also intended for infected wounds or wounds at risk of infection (Figure 1).
- Medisorb R Powder – a dressing in the form of powder made exclusively out of chitin copolyesters. The dressing is intended for dressing deep wounds (Figure 2).

The 90:10 butyric-acetic chitin copolyester (BAC 90:10) was obtained with the method using a mixture of acetic and butyric acid anhydrides in the presence of perchloric acid as a catalyst [42,43]. BAC 90:10 was a raw material for obtaining highly porous dressings. Two methods of manufacturing were developed [44]: (a) pouring BAC 90:10 solution in ethanol on a layer of solid porophor, which was later leached, and (b) using the suspension of porophor in BAC 90:10 solution, however, this method was required using a mixture of solvents of bulk density similar to the one of the used porophor. An optimal solution was used in the mixture of ethyl alcohol with chloroform as solvents. Both methods were allowed for obtaining highly-porous materials (porosity of 95–99%) of thickness up to 0.11 mm. For manufacturing of the Medisorb R Membrane and Medisorb R Ag, the method using NaCl as porophor and 3% BAC 90:10 solution in ethanol was applied. The exact manufacturing methods of the dressings and their full physio-chemical and biological characteristics (in vivo and in vitro tests on animals) are presented in other papers [44,45,46].

The Medisorb R dressings (Membrane, Powder) and Medisorb R Ag [46] are intended for dressing wounds of different etiology, including chronic wounds where the healing process is disturbed by comorbidities (e.g., cardiovascular diseases and diabetes). Those wounds are often accompanied by bacterial infections, which is the reason why one of the tested dressings demonstrated bactericidal action. It allows for the elimination of present infections and ensures protection against primary and/or secondary infection. Additionally, in order to accelerate the healing process of deep/furrow wounds, the powdered form of the dressing was designed. Regardless of the form of the dressing, each of them is intended to be placed directly on the wound. The main function of the tested dressings is evacuation of the exudate from the wound, which is possible due to the presence of microcapillaries in the dressing. It allows for the removal of the exudate from the wound into the external surface of the resorptive dressings. Due to the fact that the dressing is soluble (due to the enzyme action—chitotriosidase), there is no necessity of its change. Thanks to this, the newly-created granulation is not damaged. The products of dressings’ decomposition are mainly absorbed by the external dressing. Only a small amount may stay within the wound and it will be decomposed to N-acetyl-D-glucosamine and N-butyryl-D-glucosamine. Both substances occur naturally in the human organism and, thus, do not pose a threat to patients’ safety.

The dressings used within the clinical trials were tested in in vitro conditions where it was demonstrated that the Medisorb R dressings degrade when under the influence of the serum/plasma enzyme and that Medisorb R Ag shows bacteriostatic and bactericidal activity. There were also tests regarding irritation after multiple applications on skin, sensitization activities, healing-in process after intradermal application for up to 6 months, and the influence of the dressing on healing of skin loss of full thickness for up to 21 days [45]. It was concluded that porous Medisorb R and Medisorb R Ag dressings are biocompatible and safe materials. The tests on animals did not show any irritation or substantiation. The tested materials had a positive effect on regeneration of damaged skin tissue. Medisorb R Ag, which contains silver as an anti-bacterial agent, showed significant activity against *Staphylococcus aureus* and *Klebsiella pneumoniae* [45].

The clinical trial was conducted, according to the requirements included in the 93/42/EEC Directive related to medical devices, Act on medical devices on 20 May 2010, Regulation of the Minister of Health on 9 May 2012 on Good Clinical Practice, and PN-EN ISO 14155: 2012 "Clinical research of medical devices on humans. Good clinical practice." The dressings were evaluated in three clinical centres: Wł. Biegański Provincial Specialised Hospital, Dermatology, Paediatric Dermatology and Oncologic Dermatology Ward, Dermatology, UM Paediatric Dermatology and Oncologic Dermatology Clinic, Military Institute of Medicine, Ministry of National Defence Central Clinical Hospital, Dermatology Clinic, and in Citonet Łódź Spółka z o.o Wound Treatment Clinic.

The patients included in the clinical trial fulfilled the following inclusion criteria: age above 18, presence of chronic skin loss from ulceration resulting from venous insufficiency without the time limit for the presence of the ulceration, and wounds connected with diabetes. Another inclusion criterion was signing the informed consent form. The exclusion criteria encompassed: age below 18, pregnancy or breastfeeding, diagnosed cancer or previous clinical history of malignant cancers, intake of an immunosuppressant, anti-cancer drugs, or steroids, diagnosed allergy to crustaceans or seafood, diagnosed allergy to silver, and alarming parameters visible in morphology, urine, or biochemical tests. 

During the trial, 10 visits were planned (one visit for qualification to the trial and nine control visits). Throughout the screening visit (qualification visits), the following actions were taken: signing the informed consent form for the participation in a clinical trial, evaluation of inclusion and exclusion criteria, subject and object tests, blood and urine sampling for lab tests, and swabbing the wound. During the control visit, the following actions were taken: object and subject tests including the adverse effects, blood and urine sampling for lab tests, sampling for evaluating chitotriosidase concentration (serum) and silver concentration (urine and serum), swabbing the wounds, visual control, and measuring the ulceration, treatment of the ulceration, photographic evidence collection, application of the chosen dressing, instruction of the patient regarding the treatment of ulceration, and the application of dressings.

Sixty-five patients were subjected to qualification for the participation in the trial, out of which 36 were included in the clinical trial. Other patients, who fulfilled at least one exclusion criterion, were excluded from the next stages of the trial.

The test group consisted of 12 men and 24 women. The mean age was 65 (age range 26–96 years of age). The choice of dressings was made on the basis of evaluation of the wound, clinical signs of infection, or risk of infection. The Medisorb R Membrane dressing was used in 23 patients, Medisorb R Ag, in 15, and Medisorb R powder in 2 patients. Four patients had a different form of the dressing applied.

## 3. Results

Dressings Medisorb R Membrane, Medisorb R Ag, and Medisorb R Powder were used in clinical evaluation of the healing process of shin ulceration and/or diabetes. Thirty-six patients participated in the treatment. Medisorb R Membrane was applied in 23 patients and Medisorb R Ag was applied in 15 patients, while Medisorb R Powder was used in 2 patients (four patients were supplied with a couple of tested dressing types) (Table 1).

Table 2, Table 3 and Table 4 present the effectiveness of dressings use in various study centres.

The patients included in the clinical trial suffered from crus ulceration resulting from chronic venous insufficiency or diabetes. In one case, the ulceration was caused by the removal of a nodule. Apart from venous insufficiency and diabetes, the patient suffered the following comorbidities: hypertension, ischemic heart disease, atrial fibrillation, gastric ulcer, prostatic hypertrophy, gastroesophageal reflux disease, hyperthyroidism, myocardial infarction, bronchial asthma, diabetic retinopathy, obesity, mixed hyperlipidaemia, osteoarthritis, arteriosclerosis, cholelithiasis, osteoarthritis, thrombocythemia during follow-up, morbid obesity, Hashimoto’s disease, plaque psoriasis, haematuria, osteoporosis, pericardial fluid, post-ischemic stroke, post cardiac arrest condition, and resuscitation after brachytherapy. 

The clinical trial plan assumed a six-month follow-up period. In three cases, healing occurred after 1.5–2 months. 

During the screening visit, after the patient provided the Informed Consent Form, the patient was interviewed, the wound was assessed, physical examinations were performed, BMI was determined, blood was collected to determine glycated haemoglobin, general morphology, Enthyrocyte Sedimentation Rate, cholesterol, Prostate Specific Antigen (PSA), Cancer Antigen 125 markers (CA 125), Carcino-Embryonic Antigen (CEA), Cancer Antigen 19-9 (CA 19-9), creatinine, Aspartrate Aminostransferase (AST), and Alanine Aminotransferase (ALT). A wound swab was also taken. A general urine test and a faecal occult blood test were performed.

At the application visit, the wound was cleaned and a dressing was applied. At follow-up visits, the researcher interviewed the patient, and then, after washing the wound surface with a resorbable dressing applied, the wound was inspected, the swab was taken, and photographic documentation and planimetry were made. If the surface of the dressing was broken (the dressing resorbed), another product was applied. In addition to the above activities, physical examination of the patient was performed, blood samples were taken to determine glycated hemoglobin, general morphology, OB, cholesterol, AST, ALT, and creatinine. A general urine test, a silver urine and serum test, and a chitotriosidase test were also performed.

If the patient had adverse effects or a deviation from the norm in the results of laboratory tests was observed, the researcher made a decision about further proceedings, e.g., the inclusion of additional therapy or referring the patient to the GP.

In patients with multiple wounds—five cases—Medisorb P Plus was used as a control dressing, which followed the principle that ulcers with a smaller surface and depth were supplied with this dressing. Two patients with multiple wounds showed no improvement in ulcers supplied with Medisorb P Plus, while a reduction in the area and depth of the wound dressed with the Medisorb R Membrane (in the first patient) and Medisorb R Ag (in the second patient) were observed. One patient (described below as patient 3) healed the ulceration with both dressings, but the ulcer dressed with Medisorb R Membrane was 6.1 cm^2^ and 2 mm deep, and the ulcer covered with Medisorb P Plus had the dimensions of 0.5 cm^2^ and a depth of 0.5 mm. The healing of both wounds was noted at the same time. In two patients, no improvement in the wound condition was noted during treatment with the studied dressings or during the therapy with Medisorb P Plus dressing. The wounds were assessed based on Knighton’s classification, in which VI stages are distinguished. Stage I applies to the most superficial wounds, limited only to the epidermis, possibly to the dermis. Stages II-V describe deep changes that spread successively toward the subcutaneous tissue, fascia, and muscles, up to tendons, ligaments, and bones. Stage VI applies to extensive wounds reaching and covering even large body cavities [47].

**Figure 3:**
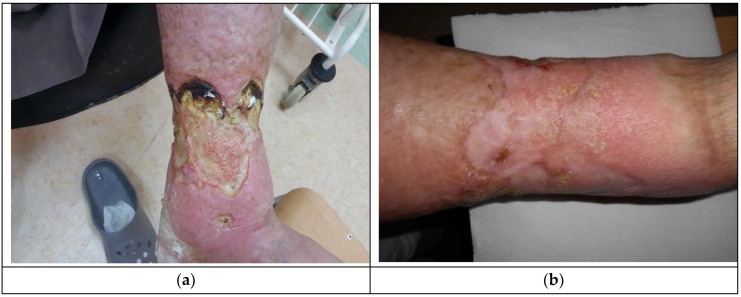
The wound treatment with Medisorb R Ag (Patient 1): (**a**) before the treatment and (**b**) after the treatment.

The decision about application of Medisorb R Ag was made in the case of a patient who suffered for a number of years from left crus ulceration resulting from venous insufficiency and who also had ischemic heart disease, circulatory inefficiency, and fixed atrial fibrillation (Patient 1). The wound exuded serous-bloody fluid and was classified as stage IV, according to Knighton. After six months, the wound surface shrank (from 238.68 cm^2^ to 27.4 cm^2^) and the wound itself became more shallow (from 4 mm to 2 mm). Figure 3a presents the wound before the therapy, while Figure 3b shows the state of the wound during the final visit.

**Figure 4:**
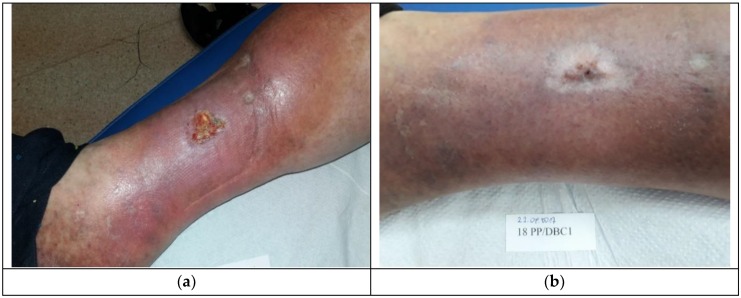
The wound treatment with Medisorb R Ag (Patient 2): (**a**) before the treatment and (**b**) after the treatment.

Patient 2, who had Medisorb R Ag applied, suffered from right crus ulceration resulting from venous insufficiency for a few years. The wound was classified as stage III according to Knighton. After two months, the patient also had gastroesophageal reflux disease. The result of the treatment was total healing of the wound (surface 5.74 cm^2^ and 3 mm in depth). It was stated that the wound was infected with *Staphylococcus aureus* and that, during the fourth visit, the wound swab culture was negative. Figure 4a shows the wound before the therapy and Figure 4b presents the wound during the last visit.

**Figure 5:**
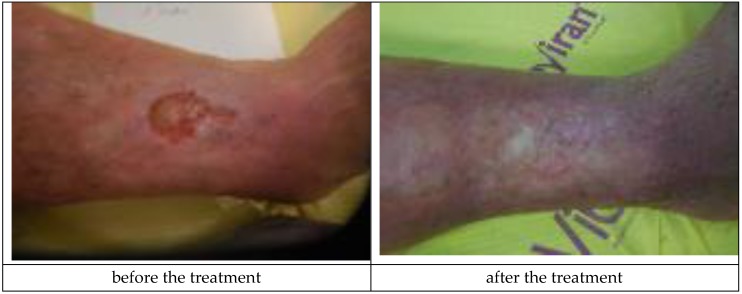
Wound treatment with Medisorb R Membrane (Patient 3).

**Figure 6:**
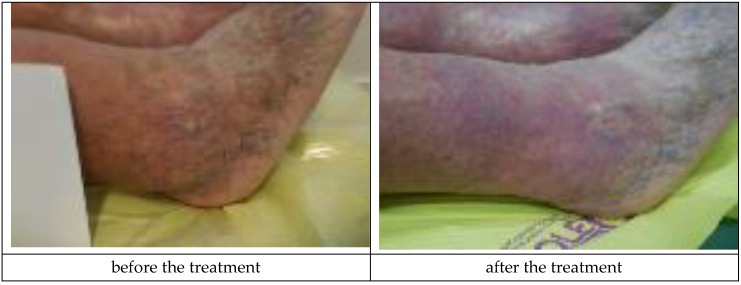
Wound treatment with Medisorb P Plus (Patient 3).

A sample patient treated with Medisorb R Membrane dressing was a patient suffering from venous insufficiency crus ulceration and chronic venous disease, hypertension, and ischemic heart disease (Patient 3). During the treatment, the patient was also diagnosed with urinary tract infection, crus rash, and features of the infection in a general urine test. It was concluded that the mentioned adverse effects were not connected with the applied dressing. At the moment of inclusion in the trial, the surface/depth of the venous ulceration treated with Medisorb R Membrane was equal to 6.1 cm^2^/2 mm (the ulceration covered the full thickness of the dermis with the subcutaneous tissue exposed, inflammation and exudation were moderate, the skin was hardened, and visible varicose veins were at stage II/III, according to Knighton). The ulceration treated with Medisorb P Plus was 0.5 cm^2^/0.5 mm. On the 47th day of treatment, it was observed that both ulcerations were healed (visible scar with no characteristics of inflammation). Figure 5 and Figure 6 present the wounds treated with Medisorb R Membrane and Medisorb P Plus before the therapy and at the final visit.

**Figure 7:**
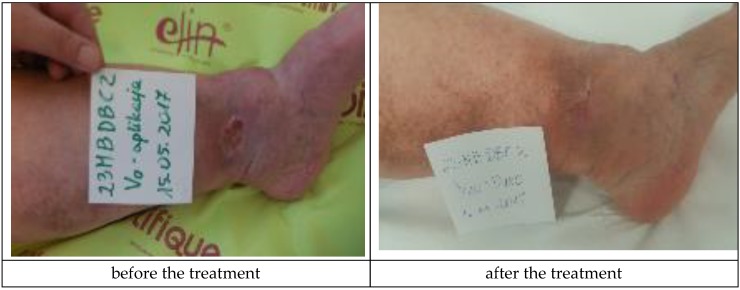
Wound treatment with the Medisorb R Membrane (Patient 4).

The Medisorb R Membrane was also applied in a patient who suffered from left crus ulceration and had the following comorbidities: hypertension, pericardial fluid (of unknown etiology), and who has suffered from a stroke (Patient 4). During the therapy, a number of deviations were observed, which were not connected with the use of the dressings (increased amount of leukocytes and erythrocytes in urine, increased leukocytes and thrombocytes in blood, and a positive test for faecal occult blood). At the moment of inclusion in the trial, the surface of the venous ulceration was equal to 10 cm^2^ and its depth was equal to 3 mm (ulceration involved full thickness of the skin with visible subcutaneous tissue, moderate inflammation and exudate, visible telangiectasia and varicose veins, and oedema of the lower leg, with the wound defined as being at the Knighton II/III stage). During the final visit at the 180th day of the therapy, the skin wound penetrated only the epidermis and was transformed to a small, superficial skin erosion of a 0.5-cm^2^ surface (moderate exudation, no inflammation, visible telangiectasia, and oedema). The applied treatment caused a significant clinical improvement of the wound. Figure 7 presents the wound during the first visit and the last visit.

**Figure 8:**
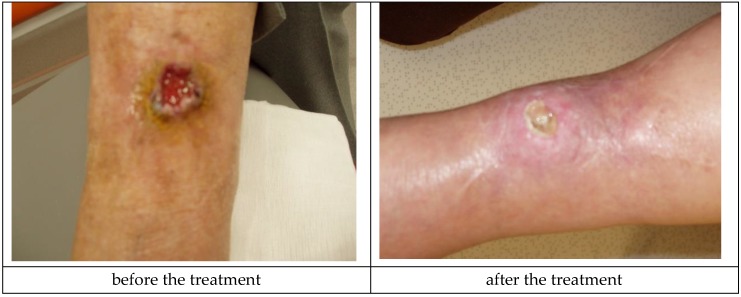
Wound treatment with Medisorb R Powder and Medisorb R Membrane (Patient 5).

Medisorb R Powder was applied in a patient with a slightly bleeding, round wound. The wound’s diameter was approximately 3 cm and its depth was 0.8 mm on the left crus (Patient 5). Additionally, as secondary dressing, the Medisorb R Membrane was applied. The wound was a penetrating wound into the subcutaneous tissue and fascia, exuding serous and blood secretions, which did not respond to using standard dressings. In Knighton’s 6-stage classification, the wound could have been described as grade III. During the therapy, the growth of granulation was observed going from the edge of the oval wound. The wound itself had a 0.8-cm deep crater-like shape. At the end of treatment (after 3 months), the wound was shallower—0.3 cm in depth. During the treatment, the depth of the wound had a significant influence on hindering the healing process in the wound’s central part, which was covered in a necrotic tissue scab. During the last visit, the Patient made a decision of terminating treatment stating that they will take care of the wound on their own. This case made it possible to positively evaluate the action of Medisorb R Powder, which had a bactericidal activity on bacterial flora, stimulated the granulation, and also limited the exudation from the wound. The action of Medisorb R Powder and Medisorb R Membrane can, thus, be evaluated positively. Figure 8 presents the wound before therapy and its state during the final visit.

During the clinical trial, the dressings were supplemented at control visits according to the trial plan. The tested dressings were not removed. If their surface was broken, another dressing was applied. The patients also obtained the dressings for home use in case the resorption process was quicker than anticipated. The number of dressings depended on the surface and depth of the wound. In some patients, additional control visits were necessary due to their physical state, which made standalone application difficult.

The deviations observed in patients’ blood and urine tests were not connected with the evaluated dressings. The dressings subjected to the clinical trial have a high safety profile. The general efficacy evaluation of the dressings stated that the products are good. In 26 patients, the surface and the depth of the wound were minimized. It has to be stated that the efficacy of the treatment also highly depended on comorbidities, especially diabetes, vein thrombosis, ischemia, low immunity, arteriosclerosis, and personal hygiene of the patients.

**Figure 9:**
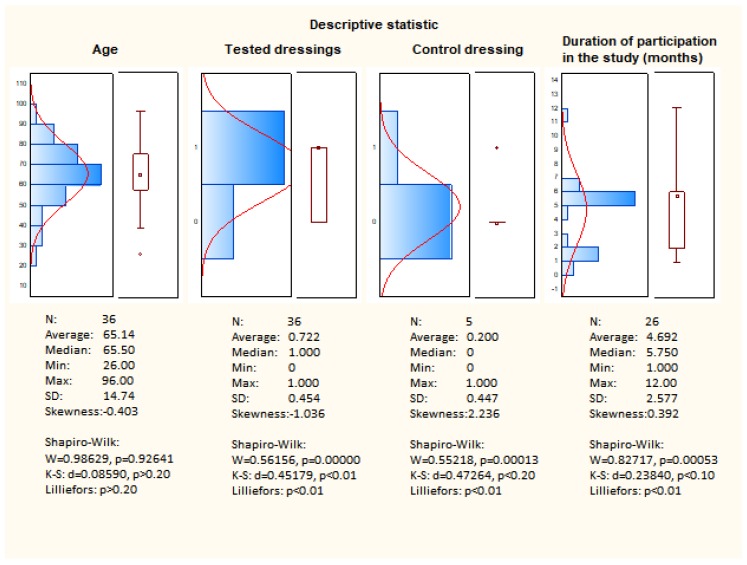
Descriptive statistics.

Figure 9 presents descriptive statistics. The clinical study evaluated the efficacy and safety of resorbable dressings (Medisorb R Membrane, Medisorb R Ag, Medisorb R Powder). The study involved 36 patients aged 26 to 96 years. The average age of the patient was 65.5 years with a standard deviation of 15 years. 

Based on the skewness, it can be stated that the age group has a symmetrical distribution. Based on the Shapiro-Wilk tests, it can be concluded that the distribution is normal, which confirms that the test group was well-chosen.

After applying the dressings (Medisorb R Membrane, Medisorb R Ag, and Medisorb R Powder) to 36 patients, improvement and healing was observed in 26 of them, and, in 10 patients, there was no improvement in the condition of the wound. The improvement constitutes 72.2% effectiveness. The obtained result is not in the form of a normal distribution, which is caused by the result evaluation scale (value 0—no improvement, value 1—improvement, healing).

Patients treated with Medisorb P Plus were assumed as a control group. This group consisted of five patients. Improvement was observed in one person and, in four patients, there was no improvement, which equals 20% effectiveness. The obtained result is not in the form of a normal distribution, which is caused by the result evaluation scale (value 0—no improvement, value 1—improvement, healing).

Patients participating in the study were treated on average for 5–6 months. The average treatment time is 4.69 months, while the median is 5.75. The lower average value compared to the median is due to the presence of outliers.

The prevailing duration of therapy is within the range of 4–6 months. The duration of participation in the study does not meet the criterion of normality distribution, which is confirmed by the W Shapiro-Wilk tests.

The significance of the differences between the tested dressings and Medisorb P Plus was demonstrated using the non-parametric Mann-Whitney U test with a continuity correction for the significance level p < 0.05 (see Table 5).

The need to use nonparametric statistics results from the lack of normal distribution for the monitored parameter.

## 4. Discussion

In Study Centre 1 in 67% of patients, the surface of the ulceration was reduced and its depth was also reduced. Total wound healing occurred in 5% of cases. Depending on the stage of ulceration (its size, depth, accompanying diseases), the improvement of the clinical wound condition could have been observed after 30–60 days. In the second study centre, the Medisorb R Membrane was applied in nine patients with diagnosed shin ulceration in venous insufficiency. Dressings were applied on the skin every 3–14 days, and were then covered with polyurethane dressing Medisorb P Plus. The therapy time and first signs of the wound condition enhancement were a very individual matter. At the moment of termination of the trial in six patients, there was a clinically significant enhancement. In two patients, a total healing of the wound was observed. In two patients, due to remission of the skin condition, the use of dressing ceased (day 47 and 54). In the rest of the patients (4), the wound condition enhancement was manifested via reduction of the surface and depth of the wound. In three out of nine patients, no enhancement was observed. In two of them, the treatment was terminated during the 60th and 30th day of the trial. In the first case, the surface of the ulceration and inflammation increased. After removal of the Medisorb R Membrane, the enhancement of the wound condition was observed. In the second case, the clinical symptoms of bacterial infection were observed. The treatment with the Medisorb R Membrane was accompanied with aimed antibiotic therapy. Nevertheless, the condition of the wound remained unchanged. Due to this reason, the patient was excluded from the trial. In the last patient, where no enhancement of the wound conditionafter application of the Medisorb R Membrane was observed, severe hygienic disregard stemming from a difficult personal situation were present. The ulceration was infected with bacteria and the state of the patient required intravenous antibiotic therapy. The ulceration was healed via skin graft. Medisorb R Ag was used in five patients with diagnosed crus ulceration resulting from venous insufficiency. The dressings were applied on skin every three to 14 days and later covered with polyurethane Medisorb P Plus dressing. The time in which the dressing was applied and the time signs of healing appeared were a very individual matter. At the time of trial termination, in three patients, there was enhancement in the wound condition manifested via a decrease in the surface and depth of the wound. One patient resigned from the trial in the 10th day of its course. In one patient, there was no clinically significant enhancement of the wound condition and the patient resigned on the 60th day of therapy. The possible cause for lack of success in this case was an infection within the wound. At the beginning, the treatment with Medisorb R Ag was accompanied with aimed antibiotic therapy. The condition was not improved and the patient resigned from the trial. In one patient, using Medisorb R Ag led to an allergic reaction to the dressing on the 158th day of the trial. Up to this moment, the healing process was smooth. The surface and depth of the ulceration were reduced. 

In Study Centre 3, the Medisorb R Membrane dressing was applied in three patients, Medisorb R Ag, also in three patients, and Medisorb R Powder in two patients. Depending on the needs, in some of the patients, one or more than one form of the dressing was used. In all patients, the reduction of the wound surface and depth was observed. 

While summing up the evaluation of efficiency and safety of the dressings, it was concluded that the Medisorb R Membrane enhances the healing process of crus ulceration. The dressings were well-tolerated by the patients and were characterized by the required efficacy. The Medisorb R Ag dressings also supported crus venous ulceration treatment. Medisorb R Ag demonstrated higher efficiency in infected wounds when compared to the Medisorb R Membrane. On the other hand, the Medisorb R Membrane was superior in treating wounds with exudate and without symptoms of bacterial infections. It was observed that the patients participating in the trials showed a stabilization in microflora of the venous ulcerations. The majority of patients tolerated the Medisorb R Ag dressing well. The highest efficiency of the dressings was observed in superficial wounds. In the case of deep wounds (depth above 7 mm), in the first stage of treatment, the wounds only became shallower. In the next stage, the wounds’ surface gradually reduced.

The efficiency of treatment is conditioned not only by the dressing material, but also by the stage of the primary disease. The healing processes may be disturbed by bacterial infections, a non-hygienic lifestyle, or comorbidities, since it was observed in patients in whom the therapy failed. In the case of lack of improvement of the wound, the advanced age of the patients and the extent of the skin lesions were also of great consequence. Lack of progress in wound healing in some patients was due to early resignation because of personal reasons. 

The patients who used the Medisorb R Ag dressing, the silver content in serum and plasma were monitored. In the collected samples, only the residual, statistically insignificant amounts of silver were traced. The detected values were as follows: for serum—0.4–2.53 µg/L and for urine: 0.027–0.214 µg/L. In the course of treatment, there were no systemic adverse reactions that may have been caused by silver ions.

The tested dressings were not removed from the wound. The application of the subsequent dressing followed the observation of the disruption of the dressing structure present on the wound (the observation was conducted every 3–14 days). The disruption of the dressing is associated with degradation of the dressing in the wound environment.

The dressings under evaluation have a high safety profile. There were no significant adverse effects of their use observed. The general evaluation of the efficiency of treatment was described as good. The Medisorb R Membrane, Medisorb R Ag, and Medisorb R Powder are intended for chronic wounds of different etiology, mainly caused by venous insufficiency and diabetes. Taking into consideration, their efficiency and easy tolerance by the organism, the dressings may be considered as one treatment method. 

The applied Medisorb P Plus control dressings are highly absorbable polyurethane dressings intended for wounds with significant amounts of exudate including crus ulceration. Apart from its function as fixing or as a protection device, the dressings also absorbed the wound exudate and purulent secretions. The Medisorb P Plus dressings in the clinical trial were used as secondary dressings placed on the Medisorb R Membrane and Medisorb R Ag, and were also applied in the case of multiple ulcerations. In the described situations, the ulceration of the smallest surface was chosen for the therapy with Medisorb P Plus dressings. The dressing change frequency was an individually fixed matter. In the case of heavily exuding wounds, the dressings were changed every 12 h. In the case of moderate or slight wounds, the dressings were changed every 24–48 hours. In six out of 16 patients, one of the ulcerations was covered only with Medisorb P Plus dressings. In one case, there was a complete healing of the wound only with the application of this dressing. The Medisorb P Plus dressings were well-tolerated by the patients participating in the clinical trial. The patients stated that their comfort was raised when taking care of the ulcerations. All this is due to high absorption properties of the dressings and protection of the wound against potential damage.

## 5. Conclusions

Created biodegradable dressings, made of butyric-acetic chitin copolyester, were evaluated in a wide spectrum of patients. The use of dressings significantly increased the wound healing process, which stems from venous insufficiency or diabetes, in the case of patients whose healing process was obstructed by comorbidities. Wound clinical condition enhancement is dependent on the patient individually, and is often observed after 30–60 days. Dressing was designed in three forms, which included a porous membrane (lack of Ag), a porous membrane with Ag, and in the form of powder. This allows for selecting an appropriate dressing type in accordance with clinical indications.

The obtained results indicate that tested dressings significantly reduce the time of the wound healing process. Medisorb R Ag, in comparison to Medisorb R Membrane, has better effectiveness in the healing process of the infected wounds. The powder form enables a dressing application in deeper wounds. Due to its unique structure, dressings drain the effusion from the wound, and restore the conditions for a proper wound healing process. Biodegradable ability, in contact with the wound effusion, eliminates the necessity of changing dressings so that it does not encroach on the newly-made granulation. The process of cell re-building occurs without interference.

The tested dressings indicate high effectiveness in the healing process of wounds of a different origin.

## Figures and Tables

**Table 1:** Number of patients who took part in the clinical trial.

Number of Patients Included in the Trial		Average Age	No. of Patients Who Used the Dressings		
			Medisorb R Membrane	Medisorb R Ag	Medisorb R Powder
F: 24	M: 12	65	23	15	2
36					

**Table 2:** Effectiveness of dressings use. Data from the study centre 1.

Effectiveness of Use of Medisorb R Membrane		Effectiveness of Use of Medisorb R Ag		
Wound condition enhancement	No enhancement	Wound condition enhancement	Healed	No enhancement
55%	45%	86%	14%	0%

**Table 3:** Effectiveness of dressings use. Data from Study Centre 2.

Effectiveness of Use of Medisorb R Membrane		Effectiveness of Use of Medisorb R Ag	
Wound condition enhancement/ healed	No enhancement	Wound condition enhancement/ healed	No enhancement
67%	33%	75%	25%

**Table 4:** Effectiveness of dressings use. Data from Study Centre 3.

General Effectiveness of Tested Dressings		
Wound Condition Enhancement	Wound Condition Enhancement	Wound Condition Enhancement
50%	50%	50%

**Table 5:** Mann–Whitney U test.

Variable	Mann-Whitney U Test (Continuity Correction) By Variable: Kind of Dressing Marked Tests are Significant at p < 0.05000									
	Rang Sum Tested Dressing	Rang Sum Control Dressing	U	Z	p-Value	Z Adjusted	p-Value	Valid N Tested Dressing	Valid N Control Dressing	2*1 Sided Exact p
Result	803.000	58.0000	43.0000	1.85260	0,06394	2.25491	0.02413	36	5	0.06261
